# Experiences of Moral Distress in Canadian Intensive Care Unit Professionals During and After the COVID-19 Pandemic: A Qualitative Exploratory Multiple Case Study in Ontario and Alberta, Canada

**DOI:** 10.1177/08850666251329828

**Published:** 2025-04-18

**Authors:** Monica L. Molinaro, Aimun Qadeer Shah, Asiana Elma, Alison Scholes, Nicole Pinto, Myles Leslie, Allison Brown, Deborah Cook, Daniel Niven, Kirsten Fiest, Elizabeth Peter, Lawrence Grierson, Meredith Vanstone

**Affiliations:** 1 Institute of Health Sciences Education, 5620 McGill University, Montreal, QC, Canada; 2 Department of Family Medicine, 3710 McMaster University, Hamilton, ON, Canada; 3 Institute of Health Policy, Management, and Evaluation, 7938 University of Toronto, Toronto, ON, Canada; 4 Health Sciences Education Graduate Program, 3710 McMaster University, Hamilton, ON, Canada; 5 School of Public Policy, 2129 University of Calgary, Calgary, AB, Canada; 6 70401 Department of Medicine, Cumming School of Medicine, 2129 University of Calgary, Calgary, AB, Canada; 7 Departments of Medicine and Health Research Methods, Evidence, and Impact, 3710 McMaster University, Hamilton, ON, Canada; 8 Department of Critical Care Medicine, Cumming School of Medicine, 2129 University of Calgary, Calgary, AB, Canada; 9 Lawrence Bloomberg, Faculty of Nursing, 7938 University of Toronto, Toronto, ON, Canada; 10 McMaster Education Research, Innovation, and Theory (MERIT) Program, 62703 Faculty of Health Sciences, 3710 McMaster University, Hamilton, ON, Canada

**Keywords:** moral distress, intensive care unit, COVID-19, qualitative multiple case study

## Abstract

**Background:** Since the beginning of the COVID-19 pandemic, moral distress among healthcare workers in the Intensive Care Unit (ICU) has garnered both media and academic attention. Moral distress has been theorized as occurring when individuals are constrained from doing what they perceive as morally right. This study sought to empirically examine the lived experiences of moral distress among clinical and administrative healthcare professionals in a sample of Canadian ICUs during the COVID-19 pandemic. **Methods:** Qualitative case study methodology was used as the overarching approach, collecting and comparing data from two distinct cases: one ICU in Ontario and one in Alberta. Data collection involved two primary sources: semi-structured interviews with staff and document review of institutional and government directives to provide contextual data. Data analysis commenced concurrently with data collection, and generated within- and across-case themes, as well as allowed descriptive accounts of moral distress. **Results:** Thirty-six healthcare workers across two sites were interviewed. Participants described three primary categories of constraints leading to moral distress. These were: 1) The rapidity and opaqueness of policy development, specifically pertaining to 2) the implementation of family visitation and treatment triage decisions, and 3) resource shortages, which reduced patient interactions, shifted professional responsibilities. Each of these constraints yielded circumstances and forced decisions that were perceived as morally wrong because they compromised care quality and outcomes. **Conclusions:** While sharing similarities with the growing literature on moral distress in the context of the COVID-19 pandemic, this study reveals new insights on how provincial and institutional policy has direct bearing on experiences of moral distress. Policies and circumstances forced ICU staff to choose between actions they considered the most right and the least wrong. Understanding these specific policy-driven constraints highlights the need for healthcare systems and processes that mitigate moral distress and sustain our health workforce.

## Introduction & Background

Moral distress, defined as “*the psycho-emotional-physiological responses of an individual who feels unable to act in a way that they believe to be consistent with deeply held ethical values, principles or moral commitments because of institutional or other constraints,*”^1,2^ has been frequently used to describe the experiences of healthcare providers during the COVID-19 pandemic. The term is, at times, conflated with experiences of burnout, stress, and general distress. Moral distress is a distinct phenomenon that encompasses several negative sequelae, including feelings of increased anxiety, exhaustion, depression, apathy, and post-traumatic stress disorder.^3–5^ While different definitions for moral distress have been posited, they tend to be united by two central tenets: 1) a constraint on action; and 2) the action constrained is one that the individual believes is ‘right’ or aligns with their moral values.^1,2,6^

The Intensive Care Unit (ICU) is recognized as an environment where healthcare professionals often encounter significant ethical challenges.^7–10^ Moral distress in the ICU has been documented in the literature since the 1980s, gaining attention in the 2000s.^11,12^ Studies have shown that professionals working in ICUs are more likely to experience moral distress compared to professionals working in non-ICU settings or who are engaged in indirect patient care.^7,13^ Fast-paced and dynamic care provision for high-acuity patients with severe, typically life-threatening illnesses characterizes ICU work.^14^ Accordingly, ICU professionals regularly confront intense decision-making scenarios, resource allocation dilemmas, and complexities in end-of-life care.^15–18^ These can include balancing professional and personal values in decision-making around admitting or discharging or withdrawing life-sustaining treatment, for example, all while navigating complex inter-professional and patient-family relationships.^19–21^ Consequently, healthcare professionals often need to quickly assess evidence and make high-stakes decisions that can have life-changing impacts on patients’ health and their family dynamics. Cumulative stress from these decisions can lead to feelings of anger, frustration, guilt, and job dissatisfaction, as well as perceived negative impacts on patient care.^19,22,23^

During the initial waves of the pandemic, ICUs worldwide were overloaded with patients severely affected by COVID-19 in addition to the high-acuity patients typically admitted to critical care.^24,25^ In the Canadian healthcare context, healthcare providers grappled with shortages of personal protective equipment (PPE).^26,27^ As the pandemic progressed, significant shortages were also realized for ventilators, hospital beds, and clinical personnel, leading to difficult decisions about allocating resources for patient care.^24,26,27^ Amidst these shortages, professionals also navigated continuously evolving provincial, institutional, and unit level policies, including restrictions on family visitation for infection control reasons.^24,28^ These evolving policies further complicated the work of ICU clinicians, adding new constraints and opportunities for moral distress to occur. Simultaneously, confusion and conspiracy theories fanned by various media increased public mistrust of healthcare professionals, exacerbating their stress, and heightening their ethical dilemmas, including navigating care for patients who did not believe in COVID-19 or the COVID-19 vaccine.^10,29^ Thus, the pandemic introduced constraints and conditions likely to foster moral distress, and presented a unique opportunity to refine collective understandings of the construct.^30,31^

The growing scholarly and media discourse on moral distress in the context of the pandemic^31–34^ has understandably focused on nurses. This attention is accounted for by their proximity to patients in daily care, which endured, although in modified fashion, during the pandemic.^30,31^ However, while nursing perspectives are essential, there is a need to integrate the experiences of other ICU healthcare and administrative professionals into our conceptualization of moral distress. For example, physicians often assume responsibility for major decisions in the ICU and faced significant pressures in decision-making the context of the pandemic.^35,36^ Other providers such as respiratory therapists, social workers, and unit managers also contribute their unique expertise as part of interdisciplinary teams in the ICU, and similarly, navigated significant pandemic-related changes to their roles in order to provide essential administration and delivery of care.^37–40^

Recognizing and understanding the experiences of moral distress among all ICU personnel is, therefore, crucial for informing strategies to better prepare the healthcare system and individual units to respond to future crises. This study examines the experiences of moral distress among administrative leaders and ICU healthcare professionals during the COVID-19 pandemic. This knowledge is crucial for surfacing the political, structural, institutional, and pandemic-specific factors that ground experiences of moral distress across the ICU.

## Methodology & Methods

This study adhered to the Standards for Reporting Qualitative Research (SRQR) guidelines.^41^ This exploratory study used Merriam's qualitative case study approach, which lends itself to “*gain[ing] an in-depth understanding of the situation and meaning for those involved.*”^42^ By situation, Merriam refers to an experience or circumstance experienced by a single unit or bounded system, such as one person, a team, a unit on a hospital floor, or a community. Merriam's qualitative case study methodology focuses on holistic description and explanation of a phenomenon, as well as promoting an understanding of context or factors that have bearing on that phenomenon. Leveraging a qualitative case study approach allowed our empirical investigation of moral distress to be mindful of the broader contexts that may bind and connect each case, such as the geographic, sociopolitical, and cultural contexts. This approach was thus chosen as we aimed to elicit answers to “*how*” and “*why*” questions related to moral distress in differing ICU contexts during the pandemic, exploring variation and diversity both within and between these environments.^43,44^

### Theoretical Perspective

This study was informed by McCarthy and Montverde's conceptualization of moral distress,^1^ which guided the development of interview guides and provided a frame through which we could analyze the descriptions of participant experiences.

### Case Boundaries and Eligibility

A “case” was defined as a hospital ICU consisting of interprofessional clinicians and staff providing critical care. Using purposive sampling,^45^ we used our professional networks to identify and approach eligible ICUs that were located within either the Canadian province of Ontario or Alberta. Ontario and Alberta were chosen as the study locations due to the jurisdictional differences in healthcare structure, delivery, and, most notably, the policies enacted in each province during the COVID-19 pandemic.^46,47^ This selection was essential for understanding how different sociopolitical healthcare contexts potentially bore influence on the constraints and subsequent moral distress that healthcare providers and administrators experienced. These cases were also well positioned to substantiate theoretical development, as they allowed us the opportunity to examine how moral distress might be shaped by various contextual factors, such as differing institutional and provincial policies.

### Sampling and Recruitment - Cases

Members of the study team with clinical expertise and networks within critical care assisted with case selection and recruitment. Conversations among critical care team members included speculation that the demography (of both teams and patients) in certain ICU regions could be heterogenous across certain axes of identity. With these considerations in mind, critical care team members suggested ICUs to the study team, and then reached out to contacts (ie, those holding administrative or leadership positions) to explore potential participation as a case in this study. The two sites that were originally suggested were the sites that ultimately agreed to be a part of this study.

### Sampling and Recruitment – Participants Within Cases

Several purposive sampling strategies were used to recruit participants within each case.^45^ Using criterion sampling, eligible participants were considered all clinicians or staff affiliated with each ICU who collaborated to provide patient care specifically within that unit, such as physicians, nurses, respiratory therapists, social workers, administrators, managers, and directors. Maximum variation sampling was also used to ensure a broad representation of perspectives. This approach aimed to recruit participants with varied clinical and administrative roles and years of experience that was also proportionate to the size of the unit's staff team. To facilitate recruitment, a case champion, identified as a practice lead or senior member of each case, was identified. Recruitment was achieved through e-mails sent by the site champion and posters (electronic and hard copy) circulated via the normal channels of internal communication (eg, e-mail listserv, bulletin board in staff room). All recruitment materials invited interested participants to contact the research team directly, so that the site champion was not aware of who elected to participate. Finally, we also leveraged snowball sampling methods, encouraging participants that had interviewed to forward recruitment materials to other individuals within their ICU who may also be interested in participating.

### Ethics

The study received all necessary approval from the Hamilton Integrated Research Ethics Board (#14646) and the Conjoint Health Research Ethics Board (#22-0986). Approval was also received from the respective ethics boards and administrations affiliated with each ICU site.

### Research Team

MM is an assistant professor specializing in critical narrative methodologies and moral distress. AQS is a Research Assistant with experience in medical education and health systems research. AE, a Research Coordinator and doctoral student, has expertise in medical education, health systems, and qualitative research. AS is a Master's student researching triage decisions during COVID-19 and moral distress. NP is a Research Associate with a focus on health policy research. ML is a researcher in health policy and practice. AB is a primary care researcher specializing in medical education and social justice. DC, a critical care physician, focuses on end-of-life care and research ethics. DN, also a critical care physician, has expertise in evidence-based critical care practices. KF is a researcher in patient- and family-centered critical care. EP, a nursing researcher, focuses on moral distress and ethical issues in community care. LG is a primary care researcher specializing in medical education and health systems. MV is a primary care researcher focusing on social and ethical complexities.

### Data Collection

Consistent with best practices for qualitative case study research, we collected multiple forms of data to systematically gather information on how a system and the individuals within it function.^43^ In this regard, we conducted semi-structured interviews with participants and collated case-relevant documents between March 2020 and March 2023.^43^

The semi-structured, one-on-one interviews included questions that elicited responses regarding their experiences of working in the ICU during the pandemic, such as instances in which they felt unable or unsure of how provide care or do the work they wanted to, or times where their work was affected by institutional or provincial policy. The interview guide was developed by consensus among the study team, which includes members with clinical (DC, DN), or scholarly expertise in moral distress (MM, ML, EP), and expertise in medical education, health policy and systems research (LG, MV). These interviews were conducted by trained research staff and graduate students (AE, AS, NP) who have experience in qualitative research. Each interview lasted between 30 and 120 min and took place either on a videoconferencing platform (ie, Zoom), telephone, or onsite at the relevant ICU. Participating individuals received a small honorarium in appreciation of their time. Interview audio files were then transcribed verbatim and de-identified. Sampling and recruitment were halted when data saturation within each case was reached to meet the objective of the study.

Our second data source, documents, were collected through a search of publicly available policies and provided by site champions. This data source offered important contextual information on institutional and governmental directives that were circulated within the respective ICUs.

### Data Analysis

Analysis of interview transcripts commenced following the first participant interview and occurred concurrently with data collection. Data analysis was a process of consolidation, reduction, and interpretation.^43^ Transcripts were read multiple times by MM, AE, and AQS, with specific attention paid to participant's recounting of experiences in which moral distress was potentially fostered, based on McCarthy and Monteverde's^1^ definition. After reading each transcript, MM, AE, and AQS used NVivo 14^48^ software to code and extract excerpts or vignettes of instances of moral distress. These particular vignettes were then further analyzed independently by at least two research team members (MM, AE, AQS), who examined: whether each experience could be considered an instance associated with feelings of moral distress, and why; whether each experience aligned with or differed from the conceptualization of moral distress (and how so); what factors within the experience were considered the “right thing” and the “constraint”; whether the participant described any emotions or reactions they felt in that moment and whether they experienced any emotions during the recounting of the event in their interview; and lastly, whether there were any other details that may have been considered relevant or important to consider for each example. Analytic memos and decisions^49^ from this exercise were recorded on a shared digital platform^49^ to maintain an audit trail, foster reflexivity, ensure transparency, and optimize rigor throughout the analytic process.^50^ The team met bi-weekly to share analytic insights, compare interpretations, discuss decisions, and resolve any discrepancies in perspectives. Any instances in which the analysts disagreed on whether an experience could be considered one in which moral distress was fostered were revisited at the next analysis meeting, so that the analysts could reflect on their and the other analysts’ responses. If the analysts did not reach an agreement after considering the vignette and each other's interpretations, then vignettes and responses were presented to two additional members of the research team (MV, LG) for their consideration. A majority consensus was achieved for each instance of moral distress that was ultimately included in the analysis.

All transcripts for one case were analyzed before moving on to the transcripts of the next case. This analytic strategy is common in collective case study research to foster a deeper understanding within each case before cross-case analysis,^51^ and allowed for a better understand the extent of the policy constraints that were presented in each context. Additionally, this approach then allowed the research team to examine general patterns, similarities, or differences between the two cases.

Documents that were collected as a secondary data source were not subjected to formal analysis but helped contextualize participants’ experiences related to the directives and policies governing provision of healthcare, visitations, vaccination, and mask exemptions as highlighted in their interviews.

## Results

36 healthcare workers working in single ICU settings in Ontario and Alberta during the COVID-19 pandemic (see Tables 1 and 2 for case descriptions) were interviewed between November 2022 and August 2023. This time period was only a few months after the seventh wave of the COVID-19 pandemic in Canada.

**Table 1.** Ontario Case Description.

Ontario Critical Care Unit (25 participants)
The critical care case from Ontario is situated within a community acute-care hospital, serving several smaller municipalities and First Nations Reserves. The hospital is loosely affiliated with a local university and has an established research center for conducting clinical trials, positioning itself as a hybrid between a community and an academic institution. The critical care unit is a level 3 unit that had less than 20 beds prior to the pandemic. During the pandemic, the unit was expanded to house 20 beds; however, this number has decreased post-pandemic. The unit is described to be intensivist-led, supported by a multidisciplinary team, and has a dedicated critical care response team (CCRT) that is comprised of specially trained CCU nurses and Registered Respiratory Therapists (RRTs) who specialize in assessing and responding to the needs of seriously ill patients. At the time of the study, the unit was supported by a mixture of full-time and part-time registered nurses, physicians, registered respiratory therapists, registered dieticians, pharmacists, and physiotherapists. Consults were often made to other allied healthcare professionals working in the hospital, such as social workers, speech-language pathologists, and palliative and psychiatry physicians. This unit was marked by critical resource shortages at the beginning of the pandemic, including a lack of personal protective equipment, medical equipment such as butterfly clips and extra tubing, and medications for patients. Provincial policies, such as the Ontario Adult Critical Care Clinical Emergency Standard of Care for Major Surge triage framework, and institutional policies, such as those governing patient visitation, deeply affected the members of this unit, as these policy decisions were seemingly often made without any consultation from the individuals who ultimately had to enact the policies.

**Table 2.** Alberta Case Description.

Alberta Critical Care Unit (11 participants)
The critical care case in Alberta is located within a large tertiary care hospital in an urban center of the province. The hospital has 1000 inpatient beds and serves a wide variety of patients from nearby cities and municipalities, as well as regions from surrounding provinces (ie, Saskatchewan, BC). The hospital is considered a level 1 trauma center with connections to the local university and medical school, and operates as part of Alberta Health Services (AHS), the province's centralized health system. As such, the unit adheres to several policies that are often enacted province-wide. The critical care unit is structured into pods consisting of just under 30 beds per pod, and is supported by a multidisciplinary team. An influx of patients during the pandemic required the unit to increase the number of beds several times, at one point expanding capacity to over 150 beds, before later reducing this to around 40 beds per pod. Healthcare providers from other hospital units or neighboring hospitals were also reassigned to the ICU to accommodate for the large-scale surge. Throughout the pandemic, various policies were considered and enacted, including the patient visitation policy. While these policies were intended to help minimize the risk of exposure and preserve personal protective equipment, they had a notable impact on the patient-clinician dynamic and the quality of care provided. In the “post-pandemic” era, the unit reportedly faced a mass exodus of staff which seemingly contributed to significantly low morale, and difficulties with recruitment for this study.

Participants included physicians, nurses, administrative managers, respiratory therapists, social workers, and registered dietitians. In both cases, there was a higher proportion of nurse participants, which was consistent with the workforce composition of each ICU. Demographic information of participants, and their identifiers for their quotes (eg, MD for physician; N for nurse) are listed in Table 3.

**Table 3.** Demographic Characteristics of Participants (N = 36).

Variable	Number of participants (%)
Gender	
Men	8 (22.2%)
Women	28 (77.8%)
Age	
20-24	1 (2.8%)
25-29	5 (13.9%)
30-34	3 (8.3%)
35-39	2 (5.6%)
40-44	7 (19.4%)
45-49	7 (19.4%)
50-54	5 (13.9%)
55-59	3 (8.3%)
60-64	1 (2.8%)
65-69	2 (5.6%)
Mean years (SD)	43.8 (11.8)
Province	
Ontario	25 (69.4%)
Alberta	11 (30.6%)
Type of Professional	
Physician (MD)	11 (30.6%)
Nurse (N)	17 (47.2)
Administrative Manager (AM)	2 (5.6%)
Registered Dietitian (RD)	1 (2.8%)
Physiotherapist (P)	1 (2.8%)
Social Worker (SW)	1 (2.8%)
Respiratory Therapist (RT)	3 (8.3%)
Years of Clinical Experience	
≤4	4 (11.1%)
5-9	4 (11.1%)
10-14	6 (16.7%)
15-19	6 (16.7%)
20-24	7 (19.4%)
25-29	3 (8.3%)
30-34	4 (11.1%)
35-39	1 (2.8%)
40-44	1 (2.8%)
Mean years (SD)	17.9 (10.4)

Participants described a range of similar challenges in relation to the provision of patient care, drawing attention to the complex and integrative influence of organizational, structural, and interpersonal constraints on their experiences of moral distress. Despite the jurisdictional differences between cases, ultimately, there were few experiential differences expressed relevant to moral distress. While they, at times, pointed to the relevance of historical issues within their respective healthcare environments, the interviews confirmed that the COVID-19 pandemic created a unique context of “*so much uncertainty*” (AB-ICU-MD01) and “*a lot of gray*” (ON-ICU-MD02), urgency “*during the pandemic information and knowledge was evolving at such a rapid pace that things were just changing so quickly*” (AB-ICU-MD01), and fear that amplified their experience of constraint that fostered experiences of moral distress.

Below, we present descriptions of experiences of moral distress as told to us by our participants. Our participants’ experiences were quite similar in nature, and thus we have organized the sections in relation to the constraints experienced with regard to: 1) the development of COVID-19 policy; 2) the entwinement of provincial and institutional policy, and then 3) resource issues and their implications for patient care.

### Visitation and Triage – Deciding Who Lives, Dies, and Says Goodbye

The most frequently reported descriptions of experiences of moral distress among all participants were associated with constraints related to provincial infection control and triage policies; specifically, as they pertained to family visitation and the possibility of deciding which patients do and do not receive resources and care.

#### COVID-19 Policy Development: Who Makes the Decisions, and Who Must Enact Them?

Some of the feelings associated with moral distress related to these policies were in relation to the lack of transparency from their institutions and provincial governments regarding “*how these [family visitation and triage] policies came to be, and on what basis they were making some of these decisions*” (AB-ICU-MD06). Participants in both provinces drew attention to how family visitation policies were developed and enforced with little to no consultation from the staff responsible for enacting them. These sentiments were summarized in a physician's discussion of the widespread policy enactment in the Alberta case:

There was no transparency around [visitation policy] that was about to come out in terms of being rolled out or kind of how those decisions were made or why they were made. It was just sort of blanket statement, this is the policy, you must follow it as of now [laughs] sort of thing with no room for negotiation. And so, a lot of those policies also were being enforced and didn’t seem to necessarily make a lot of sense in the way they were – or the policies themselves didn’t seem to always make a lot of sense. […] Those are good intentions, it's just that some of them didn’t – and the strictness and things didn’t seem to make a lot of sense. (AB-ICU-MD01)

This sentiment was also noted by participants in administrative or leadership positions who were not involved in decision-making around these policies, but who were tasked with communicating and ensuring the enforcement of these policies to their staff. One of the nurse administrators in Ontario noted that “*there were times I had to enforce a policy that perhaps I didn't 100% agree with*” (ON-ICU-A02). The participants’ recollections of these instances suggested that having to enact policies with little transparency and consultation in their development had the potential to be morally distressing.

Conversely, some physicians in both our Ontario and Alberta ICU cases were involved with the development of their province's respective patient triage policy that would be enacted during a COVID-19 surge. Those who were consulted on the development and potential enactment of these policies noted that being involved in the development process and being provided with opportunities for feedback and revision, allowed them to have a better understanding of the rationale behind the policy, and as result, their potential enactment seemed less likely to contribute to feelings of moral distress. One participant stated:

There is information circulated about that it was being developed and there was feedback asked around it as well as it was being developed. And then the final version was distributed to everyone as well so there was a lot of, yeah, transparency around it. And just because it would have created a lot of distress for people to have that just imposed on them without any input around it just because there's a lot of heightened stress and emotion around COVID that I think that was definitely an appropriate way of going about it. Otherwise, I think people would have been more resistant to it perhaps. (AB-ICU-MD01)

#### Family Visitation Restrictions: Saying Goodbye Through a Window, or Not at All

Both units in our study prided themselves on being very family-focused, which meant facilitating “*family involvement and encouraging families to be involved in kind of rounds when we go around and around on the patients and for them to be at the bedside very sort of liberal visiting hours and things like that*” (AB-ICU-MD01). Being unable to help families visit or communicate with their loved ones because of strict hospital visitor restrictions, even at the end of life, was the most common experience resulting in moral distress that participants cited. Feelings associated with moral distress were first evident to us when hearing participants’ ambivalent feelings regarding this visitation policy; many believed “*it was wrong*” for the families but simultaneously “*necessary maybe from the information we had*” (ON-ICU-A02) regarding COVID-19 infection control at the time. Even understanding the need to prevent the spread of COVID-19, participants believed that it was morally wrong to deny family members the chance to be with their loved ones, especially during their final moments: “*And at the end, your heart speaks to you because people were dying and we were preventing people from being there.*” (ON-ICU-A02).

Restrictions on direct visitation were eventually offset by the introduction of baby monitors, iPads, and walkie-talkies that providers used to connect family members with their loved ones but this solution felt just as morally distressing to staff in both ICUs, as now they were physically present and bearing witness to the distress of family members as their loved one was dying:

[…] it was horrible. We had a gentleman who was dying from COVID. He was on a ventilator not doing well, and we’re in there holding his hand as he's passing. And his teenage sons are talking through a baby monitor that we’re holding to his ear saying their goodbyes. And you’re crying your eyes out in there, and it just is – my student leaned over and said, ‘This is backwards. We shouldn’t be in here, they should be.’ (ON-ICU-N03)

Even as restrictions eased throughout both provinces in relation to social activities, there were still limits that were perceived as unnecessarily strict regarding the number of visitors or the age of visitors allowed to be in the room with the patient. One participant highlighted the stark contrast and conflict between infection control policies at the provincial level, which had different implications for their hospital compared to other public settings like a restaurant:

I remember it being this family…it was nothing to do with COVID with this family. It was a younger woman who had liver cirrhosis, had a horrific complication in which she had a tremendous neurological injury that would be that she would not survive. And they had restricted visitation on her end of life. She had two children, one was 14 and one was 16 and they said because they were under 18 that they could not come in. What I ended up doing is actually telling them to lie to the people at the front and say that they were 18. And they did lie, and they got in to say goodbye and somebody found out and then kicked them out of the hospital and said they could never return. And this, like somebody is dying [laughs] and you are restricting them from seeing their mother in their last time in life. And it's just like this is ridiculous because a person can go and sit in a restaurant. (AB-ICU-MD04)

#### Triage Policies: Who Gets to Live?

As the pandemic created a surge in demand for critical care resources, both Ontario and Alberta released triage frameworks to guide the allocation of life-sustaining resources such as ventilators. While these policies were ultimately never enacted, many participants noted that the thought of having to triage was frightening and potentially morally distressing because they would have the responsibility of declining care to some patients due to resource shortages:

I think it was pretty scary for people, I think when you started to realize what triage meant. I don’t think it really hit a lot of people. So, I think that was really the big shock of it, is that we were kind of at this point where there's potentially people we could help but they’re not going to get the help because we just don’t have the resources to help them*.* (AB-ICU-MD06).

### The Weight of Scarcity: Balancing Compassion and Constraints

The ways in which provincial policies affected each individual unit, and further, their contribution to experiences of moral distress, was further pronounced through ICU resourcing during the pandemic. Our participants noted that resource constraints, which included PPE limitations and staff shortages, forced many of them to reduce the frequency of patient interactions normally conducted in ICU settings. These constraints reportedly precipitated shifts in professional responsibilities, ultimately restricting their ability to provide what they believed to be high-quality care to patients.

#### “You’re Not Able to Help People, Even Though You Have the Knowledge, You Just Don’t Have the Resources”

Participants drew attention to how, due to limited resources - staff, PPE, and resultingly, time - patient contact became reduced and deviated from best practices in critical care. Consistent across both cases, participants recognized how policy changes, as a result of limited resources, changed their ability to conduct “*patient assessment, patient turns, mouth care, all that kind of stuff on a sedated comatose patient”* from *“every two hours*” to “*every four hours*” (ON-ICU-N02). Participants’ descriptions suggested that this reduction in contact felt morally distressing; the cutback in frequency was because of how overrun and busy their ICUs were, but participants simultaneously knew that “*the reason we do it every two hours is that's evidence-based, that's to prevent all these complications. We know that that's the standard of care. So, it didn’t feel great”* (AB-ICU-N04).

At times, infection control strategies combined with the lack of resources, contributed to negative patient outcomes, and participants’ feelings related to moral distress. As one example, participants highlighted that there were restrictions imposed on the use of medical equipment such as Biphasic Positive Airway Pressure (BiPAP) machines because they produce aerosols and could infect others being cared for or working within the ICU. These restrictions, they believed, cost several patients their lives:

I think the first six months we weren't allowed to use BiPAP whatsoever. We weren't allowed to use high flow nasal cannula, like Airvo. We weren't allowed to use certain adjuncts that we normally use to help people out, because it had to go through all these committees […] Doing code blues in the room, you'd have to move them all the way down, like on a floor. We have one code room on the floor, which is the negative pressure room to intubate and do it. So instead of doing the code blue in the room you're moving this patient, so it was a code blue on the floor, in a room, maybe down the hall […] So that's like probably four minutes […] Oh, I'm sure people die because of that. I’m sure people died, I'm sure people had more like anoxic brain injury because of that, for sure*.* (ON-ICU- RT01)

Some participants did not explicitly draw attention to the link between a lack of resources and patient care, but rather placed emphasis on how this lack of patient monitoring led to life-changing outcomes for the patients – ones that could have been prevented if staff were able to monitor their patients every two hours. Knowing that their patients’ negative outcomes could have been “picked up earlier” was morally distressing for participants:

So [an unvaccinated] woman unfortunately got stricken with bad COVID while in a late trimester pregnancy and so we had to admit her into the intensive care unit. And it was through that process somehow that she ended up having to go for an emergency C-section and did not have a viable baby…there were a few things that I wanted in the context of looking after that patient, but I was told I couldn't have because it didn't exist…Had there been continuous monitoring then I think it would have got picked up earlier*.* (AB-ICU-MD02)

In addition to the distress of reduced physical contact with patients with COVID-19, participants also drew attention to how they observed non-COVID-19 patients (or patients typically seen in the ICU) suffering from inadequate care due to resource limitations:

Yeah and I think the other side of it too is our COVID patients were really sad but the other thing is we had a lot of car accident patients, burn patients who, even though they weren’t there for COVID, were just as impacted by that. And that was also really tough. They’re there for something unrelated to what's going on and yet they can’t have that support, they can’t have anything else. And I feel like they probably didn’t get the care that they would have got if we weren’t in the middle of a pandemic…And even there was people who were waiting for heart surgery who they were supposed to go in and it was, ‘Well, sorry, we’re cancelling again because we don’t have the beds, we don’t have the ability to do it, we don’t have the resources.’ (AB-ICU-N01)

#### Shifts in Responsibilities, Care, and Outlooks

Policy changes and resource constraints during the pandemic often resulted in healthcare providers either having to expand their scope of care without any formal training or having to be redeployed to differing care environments. This often led to situations where our participants found themselves thrust into unfamiliar roles with inadequate training or preparation, and resultingly, feeling distressed about the type of care they were providing.

For instance, overflows in pediatric ICUs due to respiratory syncytial virus (RSV) in Ontario resulted in patients under the age of 18 being transferred to adult critical care environments. Nurse participants drew attention to how they and their colleagues from adult emergency departments were told to care for these pediatric patients, despite lacking the necessary expertise and familiarity with pediatric-specific protocols and medications:

With the exception of the emerge nurses who are cross-trained with pediatrics experience, they do not have pediatric experience as nurses. Their drugs are different, they're not just little adults, their care plan is different, they come with a whole different group of psychosocial, contextual issues […] literally everything about their care is dramatically different and we don't have that training […] We are trained adult ICU nurses being asked to care for patients that we have no training. So, it's like taking those other nurses and dumping them into our adult unit and telling them, have at it. They have the basic idea what to do, but no training, but that's not safe, that's not good for the nurses, that's not good [for the] patients and these are kids. (ON-ICU-N03).

Similarly, nurses who were redeployed to ICU settings from other departments were often unable to receive the requisite training prior to starting work in critical care, and one nurse recounted an emotional experience where their lack of training could have led to adverse patient outcomes:

I think my biggest example where it kind of hit me was I had a patient who coded, hard stop, we’re doing CPR, we’re doing everything. And I’ve experienced that on the unit, but on the unit we call a code and the code team comes and they do what they’re going to do, whereas in the ICU we are the code team […] I did everything I was supposed to do, but then I didn’t know what to do next […] In the moment I was like, wow, if only I knew, if only I had code training I could have maybe made a difference. And it probably wouldn’t have changed things but in the moment, I felt like it would have and I felt like if this patient had been with an ICU nurse and not with me, maybe they would have done better […] I think just in the moment you feel like I didn’t know what to do and therefore this is my fault that this happened. (AB-ICU-N01)

Overall, these accounts highlight how external constraints, predominantly related to institutional and provincial policy, as well as the rapidity and lack of transparency and support with which these policies were enacted, contributed to experiences of moral distress during the COVID-19 pandemic. These policy shifts not only affected the level of knowledge and certainty our participants had, but further had several negative implications for patient care, leaving our participants navigating the ethical implications of their actions under these ever-shifting policies. In some cases, the prolonged constraints and lack of support in participants’ ability to be the “type of [provider]” or “the type of person” (ON-ICU-N05) they wanted to be, contributed to their decision to leave the profession altogether.

## Discussion

This study presents the findings derived from 36 semi-structured interviews with healthcare workers and administrative staff who worked in ICUs in Ontario and Alberta, Canada during the COVID-19 pandemic. Findings underscore how the initial waves of the COVID-19 pandemic introduced several new constraints that severely impacted healthcare workers’ ability to provide care to their patients, which, in turn, led to experiences of moral distress. Feelings of moral distress were associated with broader provincial policies related to infection control and triage, as well as provincial and institutional decision-making regarding the use of resources including staff, medical equipment, and PPE. These factors were not just the sources of moral distress but also influenced patient care, staff well-being, and healthcare delivery.

Recently published research on this topic shows that moral distress in healthcare workers was frequently reported during the COVID-19 pandemic.^52,53^ The experiences of moral distress described by our participants are congruent with those reported by healthcare workers during the COVID-19 pandemic globally,^31–34^ particularly among ICU nurses and respiratory therapists. Specifically, our findings align with reports of moral distress related to uncertainty and being unprepared for navigating the care of patients during the pandemic, attempting to respond to new and growing information about COVID-19 in real time, trying to keep family members informed about their loved ones’ conditions, navigating visitation policy, and coping with a lack of support from management and institutions.^8,30,54–57^

While our findings are concordant with some published literature, they also offer new insights. First, perspectives from our participants highlight how broader provincial and institutional policy heavily contribute to moral distress and are further exacerbated by the perceived lack of transparency and accountability in policy decision-making processes. The findings emphasize the importance of incorporating the perspectives of healthcare workers with differing roles in institutional and provincial policymaking, particularly as healthcare workers, and ultimately the patients, bear many of the downstream consequences of these broader policy decisions.^58–60^

Our findings further support the relationship between moral distress and structural and systems issues.^61^ A longstanding body of research has established the relationship between moral distress and the structures and constraints of healthcare outside of pandemic circumstances.^62–65^ Emerging research from the COVID-19 pandemic demonstrates the amplification of these structural constraints.^8–10,26,32,57,66^ Specifically, in our study and others, we learned how moral distress affects healthcare workers who are left to compensate for system-level issues (eg, corporatization of healthcare resulting in understaffing, budget limitations, and commodification of patients) when asked to accommodate poor planning, increased workloads and expanded scope of responsibilities with little training, compensation, or resources. These healthcare workers are then left in a precarious position, where structural and organizational issues impede their ability to make decisions that align with their professional and moral values, constraining them from doing what they believe to be best for their patients. Our participants, in sharing experiences, expressed feeling constrained in their ability to act, or perceptions that long policies of austerity in healthcare created situations where they had to decide on the most morally right, or least morally wrong, course of action. Further, these constraints, which often led to redeployment, or expanded roles without prerequisite training, were perceived to be associated with adverse patient outcomes and exacerbated feelings of moral distress. For some, these experiences led to the decision to leave their profession altogether. While previous research has demonstrated how the pandemic disrupted and changed provider duties of care, particularly for nurses,^9,66^ our findings draw a direct link between such disruptions and the experience of moral distress. More specifically, the ways in which healthcare provision is currently structured is both exacerbating and facilitating the increased frequency of moral distress, and contributing greatly to healthcare worker attrition.^31,67^ This calls for the urgent re-examination of healthcare structuring for health human workforce retention and future pandemic preparation.

Finally, it was evident that while the examples discussed by our participants aligned with McCarthy & Monteverde's^1^ guiding definition of moral distress, they also presented additional features that are not encompassed within this conceptualization. Of note, participants had experiences resembling moral distress when they felt constrained from taking actions that they felt would be doing ‘most right’ by *another*, whether that ‘another’ was an actual or hypothetical person (such as a patient, family member of a patient, or colleague), a group of people, or their own idealized identity as the person and healthcare worker they aspired to be.

Further, our data highlighted that moral distress does not only manifest when one is constrained from choosing the one action that they feel is right, but also when one encounters moral dilemmas.^68^ Specifically, a moral dilemma is an instance when:

an agent regards herself as having moral reasons to do each of two actions, but doing both actions is not possible […] The crucial features of a moral dilemma are these: the agent is required to do each of two (or more) actions; the agent can do each of the actions; but the agent cannot do both (or all) of the actions. The agent thus seems condemned to moral failure; no matter what she does, she will do something wrong (or fail to do something that she ought to do).^68^

For example, our participants described experiences in which they too were condemned to moral failure, particularly when they encountered circumstances with multiple options in which they were constrained in their ability to do what they believed was *most* right, or *least* wrong. For instance, infection control policies, particularly in relation to family visitation, presented two potentially right (or wrong) options, or actions, for our participants. In one instance, following the infection control policy (ie, not allowing family visitations) could be perceived as most right, or least wrong – upholding this policy ensured that visitors with unknown COVID status were not entering the ICU and potentially infecting staff and their critically ill patients, but doing so also meant that families were prevented from saying goodbye to their loved ones, or seeing them for one last time when their loved one was dying. Simultaneously, allowing family visitation, and going against infection control policy, could be perceived as most right, or least wrong – allowing families to visit their loved ones gave them the opportunity to see their loved one, say goodbye, and have some sense of closure before their loved one died, but also meant that staff and other patients on the unit could potentially be infected with COVID, causing worse health status in patients, increased limitations on supplies, staff, time, and resources, and increased risk of COVID spread outside of the ICU. This signaled to us that moral distress is not experienced solely in relation to being constrained in doing what is right. Rather, moral distress can also manifest through moral dilemmas, when moral actors must choose between two options that are two-sided in their orientation of moral rights and wrongs. While some actors may choose what they believe is the least wrong of the options presented, others may choose what they believe is most right. Regardless of which option was chosen, participants clearly and consistently expressed that the circumstances of the COVID-19 pandemic placed ICU staff in an impossible situation in which they were *made* to choose, condemning them to experiencing moral distress for not being able to uphold both options.

These experiences of moral distress were often accompanied by feelings of guilt, frustration, and despondency. While some participants highlighted singular, stand-out moments that prompted feelings of moral distress, others experienced an accumulation of morally distressing experiences as the pandemic continued. Descriptions of frequent, continued, or heightened moments of moral distress related to the rapid and iterative implementation of institutional and provincial policies signalled to us that the phenomenon of moral distress is not confined to specific instances but rather can be experienced in a manner that grows over time. These feelings and experiences resemble those of moral residue, or “that which each of us carries with us from those times in our lives when in the face of moral distress we have seriously compromised ourselves or allowed ourselves to be compromised.”^69^

We posit that a new examination into the features of moral distress is warranted. Indeed, additional research examining experiences of moral distress during the pandemic has similarly called for a re-examination of the concept, as COVID-19 not only exacerbated historical issues within healthcare systems, but brought forward additional constraints and new scenarios that may have given rise to experiences of moral distress not previously considered.^30^

## Strengths and Limitations

Two strengths of our study are that it generates new understandings of moral distress within the context of the COVID-19 pandemic; and that it elicits perspectives from a range of healthcare workers, including physicians, nurses, physiotherapists, administrators, registered dieticians, social workers, and respiratory therapists. Rich, empirical data from the perspective of those on the frontlines of ICUs allows us to draw links between broader structural and institutional factors and individual experiences, contributing to novel understandings around the experiences of moral distress in critical care.

This research is bound by the historical and geographical contexts in which it was conducted. While the findings are not intended to be generalizable to all clinical disciplines, ICUs, or providers, they may have less salience for those working in different settings. Additionally, the findings of this study may have less relevance in clinical environments, or geographic regions, that were differently impacted by the COVID-19 pandemic. Participants were interviewed several months after the height of the pandemic, and so they may have limited recall or may have re-contextualized early pandemic experiences.

## Conclusion

This multiple case study of healthcare and administrative providers in two Canadian ICUs offers empirical insight into the ways in which the COVID-19 pandemic was an extreme, unique circumstance that generated new insights on experiences of moral distress in these workers. It documents the sources of moral distress as the urgent policy development that was shrouded by a lack of transparency; the ways in which these policy changes, particularly family visitation and triage policies, left healthcare workers in situations feeling like they could not do the right thing; and resource shortages that limited patient interactions and shifted professional scopes of care and responsibilities.

Our findings suggest that moral distress is experienced not just as a single instantaneous moment in which someone is constrained from doing what is right, but that moral distress can manifest through several situations over longer periods of time in which one is stuck within a moral dilemma, trapped in positions where they must choose options they believed were most right, or least wrong. Knowing that policy development and implementation were significant sources of constraint draws attention to how health care structuring must be changed to better support the well-being of healthcare workers.
